# Prenatal Diagnosis of Malformations of Cortical Development: A Review of Genetic and Imaging Advances

**DOI:** 10.3390/biomedicines14010107

**Published:** 2026-01-05

**Authors:** Jinhua Hu, Xiaogang Xu, Ping Jiang, Ruibin Huang, Jiani Yuan, Long Lu, Jin Han

**Affiliations:** 1 Guangzhou Women and Children’s Medical Center, Guangzhou Medical University, Guangzhou 510623, China; hujinhua0701@163.com (J.H.); xiaogang410428700@163.com (X.X.); huangruibin96@hotmail.com (R.H.); y1964697@163.com (J.Y.); 2 Dongguan Kanghua Hospital, Dongguan 523080, China; jp677699@163.com; 3 School of Information Management, Wuhan University, Wuhan 430072, China

**Keywords:** malformations of cortical development, prenatal diagnosis, chromosomal microarray analysis, whole exome

## Abstract

Malformations of cortical development (MCD) are a group of neurodevelopmental disorders caused by abnormalities in cerebral cortex development, leading to conditions such as intellectual disability and refractory epilepsy. The prenatal phenotypes of MCD are complex and non-specific, complicating accurate diagnosis and prognosis assessment. Genetic testing, particularly chromosomal microarray analysis (CMA) and whole-exome sequencing (WES), has become an important tool for prenatal diagnosis. This review synthesizes current research on prenatal MCD, focusing on the integration of imaging and genetic diagnostic strategies based on the biological foundation of cortical development and the classification system of MCD. Prenatal MCD phenotypes show significant developmental stage clustering, with proliferation-phase abnormalities (62.9%) being the most common and microcephaly as the core phenotype. Genetic studies have revealed a high degree of genetic heterogeneity in MCD, with etiologies encompassing chromosomal abnormalities and a wide range of single-gene mutations. These mutations are clustered by phenotype: microcephaly is associated with neuronal proliferation/DNA repair genes; macrocephaly is driven by genes in the PI3K-AKT-mTOR and RAS-MAPK signaling pathways; and gyral and sulcal abnormalities are closely linked to microtubule-associated genes and migration pathways. De novo mutations account for the majority of pathogenic genetic alterations identified in MCD (50.6%); up to 75.1% of pathogenic mutations cannot be detected by routine prenatal screening. Based on this, the review emphasizes that for fetuses with suspected MCD, NGS, with WES at its core, plays an increasingly important role in achieving early and accurate prenatal diagnosis. Future research should prioritize the advancement of integrated diagnostic methods and large-scale cohort studies to further elucidate genotype–phenotype associations.

## 1. Introduction

Malformations of cortical development (MCD) are a group of neurodevelopmental disorders caused by abnormalities in the development of the cerebral cortex [1,2]. The main clinical features include intellectual disability, developmental delay, and refractory epilepsy, which severely affect the neurodevelopmental progression and quality of life of affected children [3,4]. The pathogenesis of MCD is complex, typically involving disruptions at key stages of cortical formation, including the proliferation of neural precursor cells, neuronal migration, and late-stage cortical organization [1,2,5]. The clinical phenotypes vary at different developmental stages, leading to significant phenotypic and etiological heterogeneity in MCD. The long-term prognosis differs significantly, ranging from asymptomatic cases to severe neurological deficits [3,4,6,7].

Traditionally, the diagnosis and classification of MCD primarily relied on postnatal imaging and pathological examination [5]. However, fetal brain developmental abnormalities are often challenging to detect accurately at early stages using conventional imaging techniques such as ultrasound [8]. With the advancement of fetal neurosonography and fetal MRI technology, more MCD cases are being discovered and diagnosed prenatally. Despite this, the imaging phenotypes of fetal MCD are often complex and non-specific, making it difficult to accurately identify potential molecular causes, assess prognosis, and predict recurrence risk based solely on imaging [9,10,11,12]. Therefore, genetic testing plays a crucial role in the precise diagnosis of prenatal MCD. In recent years, the rapid development of molecular genetics, particularly chromosome microarray analysis (CMA) and next-generation sequencing (such as whole-exome sequencing (WES) and whole-genome sequencing (WGS)), has significantly enhanced the diagnostic capabilities for prenatal MCD [13,14,15,16,17,18,19]. These technologies enable the identification of pathogenic variants and molecular pathways underlying MCD, opening up new possibilities for early intervention and genetic counseling. However, the genetic spectrum, genotype–phenotype associations, and the optimal genetic diagnostic strategies for prenatal MCD are still major challenges facing the field.

This review aims to integrate the current research on prenatal MCD, systematically explaining its biologically based classification system, integrated diagnostic strategies combining imaging and genetics, prognosis evaluation methods, and multidisciplinary management approaches, and further clarify the possible causes and risk factors that may contribute to the wide variation in clinical phenotypes. thereby providing clinicians with forward-thinking and practical guidance for managing such complex conditions.

## 2. Biological Basis of Cortical Development and MCD Classification System

Cortical development is a highly coordinated and precisely regulated complex process, involving the proliferation of neural progenitor cells, neuronal migration, and late-stage cortical organization [5,20,21]. Each stage requires precise temporal and spatial regulation, and any disruption at these stages can lead to cortical malformations. Based on the developmental stage most affected, the internationally recognized classification system categorizes MCD into three main types: proliferation-phase abnormalities (Type I), migration-phase abnormalities (Type II), and post-migration-phase abnormalities (Type III) [1,2,5].

### 2.1. Proliferation Phase

The proliferation phase is a key stage during which neural progenitor cells divide, proliferate, and differentiate into neurons and glial cells. This phase determines the basic size and shape of the cerebral cortex, with the number of neural progenitor cells directly dictating the final number of neurons [2,4]. Therefore, abnormalities during the proliferation phase often led to significant changes in head size, with typical manifestations including microcephaly and megalencephaly. Microcephaly is typically caused by insufficient proliferation or premature death of neural progenitor cells, characterized by a significantly smaller head circumference in the fetus or newborn, often accompanied by intellectual disabilities, cognitive impairments, and developmental delay [22]. In contrast, megalencephaly results from excessive proliferation of neural progenitor cells or an imbalance between proliferation and apoptosis, leading to abnormally large head size and excessive cortical thickening. Disruptions during the proliferation phase ultimately led to cortical hypoplasia or structural abnormalities, with significant changes in the number of neurons, profoundly affecting the overall function of the nervous system [23,24].

### 2.2. Migration Phase

The migration phase is a crucial process in which neurons migrate from the germinal matrix to specific locations within the cerebral cortex. Abnormalities during this phase hinder proper neuronal positioning, leading to cortical structural disruption. Typical phenotypes include lissencephaly and pachygyria [1,3,20]. These malformations are typically associated with severe intellectual disability and refractory epilepsy. Lissencephaly is the most representative migration-phase abnormality, characterized by the lack of normal gyral and sulcal development in the cerebral cortex, presenting a smooth cortical surface. This malformation severely impairs cognitive and motor functions, especially in genetic cases, often accompanied by early-onset seizures [11]. Similar to lissencephaly, pachygyria also falls under migration-phase abnormalities, characterized by a reduced number of gyri and an increased width of the remaining ones. Although the cortex retains some gyral structure, its morphology is significantly altered, often accompanied by developmental delay and cognitive impairments. The molecular mechanisms of migration-phase abnormalities involve deficiencies in cell signaling, extracellular matrix defects, or cytoskeletal dysfunction, which hinder the migration of neurons to their target locations [11,25].

### 2.3. Post-Migration Phase

The post-migration phase refers to the stage where neurons, after reaching their target locations, begin fine cortical layering and organization. Abnormalities in this stage affect the final cortical structure, leading to layering disorganization. Common abnormalities in the post-migration phase include focal cortical dysplasia (FCD) and polymicrogyria (PMG) [6,26,27,28]. These abnormalities are challenging to detect during the fetal stage but typically present as epilepsy or other neurological deficits after birth. FCD typically presents as abnormal development in localized areas of the cerebral cortex, leading to disordered cortical layering. It is commonly seen in patients with epilepsy, and MRI imaging may show underdeveloped local gyri or abnormal layering [27]. PMG is characterized by an excessive number of small gyri with inadequate separation between them. This abnormality often coexists with lissencephaly and ventricular abnormalities and is frequently associated with cognitive impairments and seizures [26,28]. Post-migration abnormalities are often caused by disruption in neuronal signaling, extracellular matrix abnormalities, or defects in glial cell development, which ultimately impair the normal structure of the cerebral cortex.

### 2.4. Complex MCD

Complex MCD has gained increasing attention in the study of the etiology of MCD. These malformations manifest as abnormalities from multiple developmental stages interacting, forming complex phenotypes across stages [2,10]. For instance, some gene mutations can simultaneously disrupt both the proliferation and migration processes, resulting in microcephaly associated with lissencephaly or pachygyria. The emergence of complex MCD typically indicates the involvement of pathogenic mechanisms that affect multiple molecular pathways, often accompanied by multiple developmental disorders, thus significantly increasing the complexity of diagnosis. MCD is often associated with the interaction of multiple signaling pathways, such as the abnormal activation of the PI3K-AKT-mTOR pathway, which may result in megalencephaly and polymicrogyria [29,30,31], while dysfunction in the microtubule cytoskeleton can lead to neuronal migration defects and lissencephaly [32,33]. The interaction of multiple gene mutations together forms the complex pathogenic network of cortical malformations.

Understanding the underlying developmental processes and their associated genetic mechanisms not only helps elucidate the etiology of MCD but also provides a theoretical basis for prenatal diagnosis and genetic counseling. With the development of imaging techniques, the detection rate of complex MCD has been increasing, indicating that future research should focus more on the interactions between multiple developmental stages and their impact on brain development [2].

## 3. Systematic Description and Analysis of Included Prenatal MCD Studies

**Figure 1:**
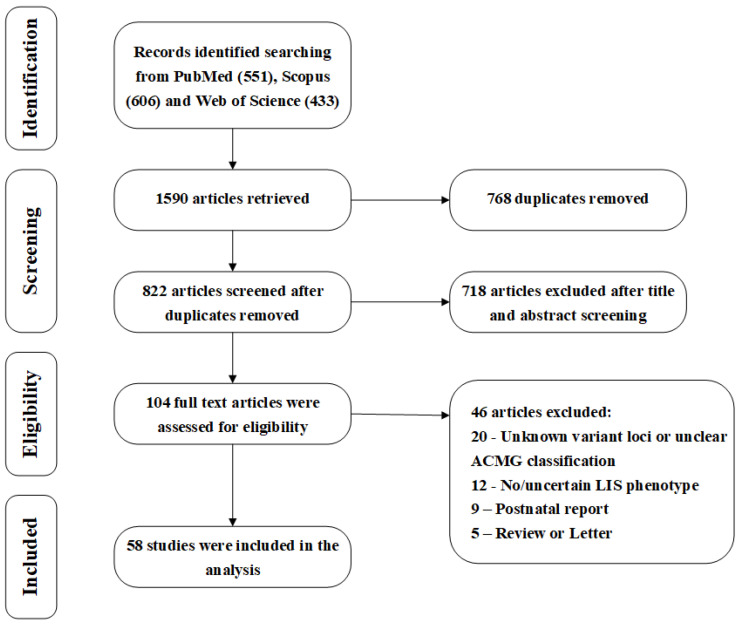
PRISMA flow diagram.

To systematically elucidate the characteristics of prenatal MCD, this study searched PubMed, Scopus, and Web of Science databases up to 17 October 2025 (Supplementary File, S1 and S2), initially obtaining 1590 articles. After excluding 768 duplicate articles, the remaining 822 articles were screened by title and abstract. Subsequently, 104 articles were fully reviewed and evaluated, and the following criteria were applied: (1) the study subjects were prenatal MCD cases; (2) high-throughput genomic technologies such as CMA, WES, and WGS were used; and (3) detailed imaging data and their correlation with genetic findings were provided. Ultimately, 58 studies met the inclusion criteria and were used for qualitative and quantitative analysis in this review [13,14,15,16,17,18,19,23,25,34,35,36,37,38,39,40,41,42,43,44,45,46,47,48,49,50,51,52,53,54,55,56,57,58,59,60,61,62,63,64,65,66,67,68,69,70,71,72,73,74,75,76,77,78,79,80,81,82]. The detailed literature screening process and exclusion reasons are shown in Figure 1. All included cases underwent rigorous prenatal imaging, genetic evaluations, and clinical analysis. Through a systematic review of these cases, this study aims to explore the phenotypic spectrum, genetic etiology, and clinical significance of prenatal MCD.

**Figure 2:**
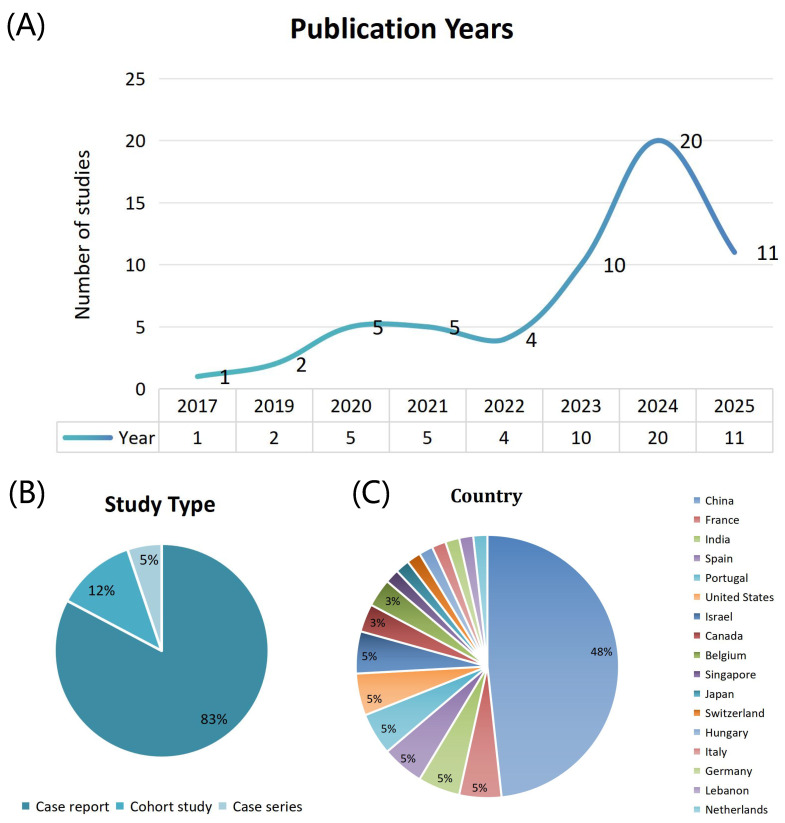
Trends in fetal malformations of cortical development research across publication years, study types, and countries. (**A**) Number of publications across years. (**B**) Distribution of study types. (**C**) Distribution of publications by country.

The basic characteristic analysis of the 58 studies included is shown in Figure 2. The studies included in this review were published between 2017 and 2025, with a significant increase in the number of publications in 2023 (10 studies), 2024 (20 studies), and 2025 (11 studies), collectively accounting for 70.7% (41/58) of the total, reflecting the growing attention to prenatal MCD genetic testing with the advancement of high-throughput genetic technologies. In terms of study types, case reports dominate, with 48 studies (82.8%), followed by cohort studies (7 articles, 12.1%) [13,14,15,16,17,18,19] and case series (3 articles, 5.2%) [62,63,73]. This distribution suggests that most of the current evidence in the field comes from small-sample case reports, which, while valuable for early identification and diagnosis, highlight the need for more large-scale prospective cohort studies to validate and deepen findings in terms of evidence rigor and generalizability. In terms of geographic distribution, China leads with the highest number of studies (28 studies, 48.3%), followed by a wide representation from North America (3 studies from the US, 2 from Canada), Europe (3 studies from France, Portugal, and Spain, 2 from Belgium), and other regions of Asia (3 studies from India and Israel). This distribution highlights the significant contributions of Chinese scholars in this field and underscores the importance of global multicenter collaborations for improving diagnostic accuracy and the generalizability of conclusions regarding this complex disease.

## 4. Prenatal Imaging Features and Clinical Correlation with MCD

Based on a systematic analysis of 237 prenatal diagnostic cases from 58 studies, this study reveals the specific distribution patterns and phenotypic characteristics of fetal MCD (Table 1). Overall, cortical developmental abnormalities show a distinct stage-based clustering. Proliferation-phase abnormalities (Type I) dominate, comprising 149 cases (62.9%), with the primary phenotype being microcephaly (107 cases). This suggests that insufficient neuronal generation is the most common developmental disorder mechanism during the prenatal phase. Migration-phase abnormalities (Type II) account for 61 cases (25.6%), primarily presenting as gyral morphological anomalies, including lissencephaly, pachygyria, and other gyral anomalies, with 48 cases making up the main phenotype for this phase. Polymicrogyria and heterotopia, among other specific migration disorders, are comparatively less common. Remarkably, complex MCD (Type I + II) accounted for 24 cases (8.4%), all exhibiting a combination of head circumference abnormalities (microcephaly, macrocephaly, or megalencephaly) with gyral anomalies. This pattern strongly indicates the presence of a complex genetic etiology that simultaneously affects both neuronal proliferation and migration processes. In contrast, Type III abnormalities, representing cortical organization defects, are very rare, with only 3 cases of focal cortical dysplasia identified. This may be related to the early prenatal diagnostic window, where such late-stage developmental abnormalities have not fully manifested.

The distribution patterns of these phenotypes provide clear guidance for prenatal genetic diagnostic strategies. Microcephaly, as the most common single phenotype (accounting for 45.1% of total cases), should be a primary focus in prenatal genetic testing, with priority given to genes related to primary microcephaly. The dominance of gyral abnormalities in the migration phase highlights the need for targeted analysis of neuronal migration-related genes, such as *PAFAH1B1*, *DCX*, and *TUBA1A*. For fetuses with complex phenotypes, the features of cross-stage abnormalities indicate that clinicians should adopt a more comprehensive genetic evaluation plan, combining CMA and WES to explore pathogenic variations that may affect multiple developmental pathways. The prenatal phenotypic spectrum depicted in this study provides key evidence for understanding the etiology of fetal brain developmental abnormalities and optimizing clinical diagnostic pathways.

## 5. Genetic Findings and Technical Analysis of Prenatal MCD

### 5.1. Chromosomal Abnormalities and Their Diagnostic Value in MCD

In 57 cases that received CMA, we identified a wide spectrum of genomic imbalances, including deletions, duplications, aneuploidy, and complex rearrangements, highlighting the comprehensive diagnostic value of CMA in prenatal genetic testing. Among them, 9 cases of numerical chromosomal abnormalities were detected: triploidy (2 cases), trisomy 16 (2 cases), trisomies 13, 14, 21, 22, and X monosomy (1 case each). It is noteworthy that 4 cases were mosaic, and the severity of their phenotype was related to the proportion of abnormal cell lines. All these cases exhibited microcephaly, reflecting the severe impact of global genomic dosage imbalance on brain development.

Abnormalities of chromosome 17 were particularly notable in CMA testing, with 12 cases of 17p13.3 deletions identified (1 case identified by WES), making it the most common single genomic imbalance. This region contains the *PAFAH1B1* gene, which is closely linked to lissencephaly, and its deletion directly causes severe neuronal migration defects. Phenotypically, 17p13.3 deletions were observed not only in gyral abnormalities (such as lissencephaly, 10 cases) but also in isolated microcephaly (2 cases). Another important abnormality of chromosome 17 is the 17q12 duplication (4 cases), all of which presented with microcephaly. This region contains several neurodevelopmental genes, and its duplication may disrupt normal cortical development through gene dosage effects. Moreover, a 17q22 deletion detected in a microcephaly case further broadens the role of chromosome 17 in cortical development.

Additionally, 7 cases of 4p16.3 microdeletions were identified, all presenting with microcephaly, which aligns with the typical characteristics of Wolf–Hirschhorn syndrome. This region contains several key genes, including *WHSC1* and *WHSC2,* and their deletion can lead to severe neurodevelopmental disorders. One case showed a 4p16.2 deletion, suggesting the presence of key genes in this region that may be sensitive to gene dosage. The consistency of the phenotypes in these cases emphasizes the essential role of the 4p16 region in brain growth regulation. Other notable genomic imbalances include 2 cases of 16p13.3 deletions (associated with Rubinstein–Taybi syndrome, which is typically characterized by microcephaly and distinctive facial features) and 2 cases of 7q11.23 deletions (1 detected by WES), linked to Williams syndrome (although this syndrome is usually not characterized by microcephaly, two studies included in this review reported cases of microcephaly combined with fetal growth restriction (FGR), expanding its phenotypic spectrum). We also identified several complex genomic rearrangements, including one case of 2p25.3 duplication combined with 9p deletion and one case of 1q43-44 deletion combined with 4p16.3 duplication. These complex rearrangements often lead to severe phenotypes, reflecting the collaborative role of multiple genomic regions in cortical development. Notably, such rearrangements often involve changes in the dosage of multiple genes, which may impact brain development by disrupting gene regulatory networks.

### 5.2. Molecular Mechanisms and Functional Clustering Analysis of Cortical Development-Associated Genes

In 180 cases detected by NGS, we found that MCD exhibited a high degree of genetic heterogeneity. In terms of technical distribution, 175 cases (97.2%) utilized WES, while 2 cases used WGS and 3 used targeted panel sequencing. This distribution reflects the central role of WES in current prenatal genetic diagnosis, as it strikes a good balance between high throughput and cost-effectiveness. It is noteworthy that WES not only successfully identified single-nucleotide variants and small insertions/deletions but also detected 3 larger segment deletions (13q14.2q32.1, 7q11.23, and 17p13.3) through copy number variants (CNV) analysis, highlighting its comprehensiveness in detecting genomic variations.

#### 5.2.1. Genetic Basis of Microcephaly: Primary Microcephaly Gene Cluster

As the most common MCD phenotype, the genetic foundation of microcephaly primarily focuses on the gene network involved in neuronal proliferation. Analysis of 180 NGS cases showed that genes related to primary microcephaly form a highly heterogeneous genetic spectrum. Among these, the *ASPM* gene is the most prominent (*n* = 13). These variants not only cause isolated microcephaly but also appear in compound phenotypes combining microcephaly with gyral abnormalities. Variants in centrosome-related genes like *MCPH1*, *WDR62*, *CEP135*, and *CDK5RAP2* further validate the critical role of centrosome function in neural progenitor cell proliferation. We also identified a range of gene mutations associated with DNA replication and repair, including *NIPBL* (4 cases), *PNKP*, *SMC3*, *TREX1*, etc. These gene defects indirectly affect brain development by causing genomic instability. Notably, mutations in the *NIPBL* gene, a chromatin cohesion protein loading factor, are typically linked to Cornelia de Lange syndrome, and our findings broaden its neurodevelopmental phenotype spectrum. Additionally, mutations in RNA processing genes (such as *EFTUD2* [3 cases] and *RNU4ATAC*) and cell metabolism-related genes (such as *PDHA1* [2 cases] and *SASS6*) together form the complex genetic network underlying microcephaly.

#### 5.2.2. Molecular Mechanisms of Macrocephaly: Dominant Roles of PI3K-AKT-mTOR and RAS-MAPK Pathways

The genetic foundation of macrocephaly mainly involves two core signaling pathways: PI3K-AKT-mTOR and RAS-MAPK. Among these, abnormalities in the PI3K-AKT-mTOR signaling pathway predominate, with 22 related variants identified, involving genes such as *PTEN* (8 cases), *PIK3CA* (3 cases), *MTOR* (4 cases), *PIK3R2* (4 cases), *AKT1* (2 cases), and *AKT3* (1 case), forming the major genetic basis of macrocephaly and megalencephaly. *PTEN*, as a negative regulator of this pathway, loss-of-function mutations lead to abnormal activation of the pathway and are a major cause of isolated macrocephaly. It is noteworthy that *PTEN* mutations were extensively detected through WES and confirmed by a specific megalencephaly and overgrowth syndrome panel, verifying its crucial role in cell growth regulation. These variants exhibit a diverse range of phenotypes, from isolated megalencephaly to complex compound phenotypes, reflecting the critical role of this pathway in regulating various developmental stages. Variants in the RAS-MAPK signaling pathway are also significant, with 23 cases identified, 16 of which involve mutations in the *FGFR3* gene. This finding broadens our understanding of the role of craniosynostosis-related genes in cortical development. Furthermore, mutations in *PTPN11* (3 cases), *HRAS* (1 case), and *NSD1* (3 cases) further expand the genetic spectrum of this pathway. Notably, in contrast to PI3K-AKT-mTOR abnormalities, which lead to compound phenotypes, RAS-MAPK pathway mutations primarily cause isolated macrocephaly, highlighting the specificity of different signaling pathways in phenotype expression. Variants in the cell cycle positive regulator gene *CCND2* (3 cases) lead to excessive neuronal proliferation by promoting the G1/S transition, constituting another important mechanism of macrocephaly.

#### 5.2.3. Genetic Network of Gyral Abnormalities: Essential Genes in Neuronal Migration and Microtubule System

Gyral abnormalities due to neuronal migration disturbances (including lissencephaly, pachygyria, and polymicrogyria) have a genetic basis primarily involving the microtubule cytoskeleton system. Microtubule-associated genes form the core genetic factors, such as *TUBA1A* (2 cases), *TUBB2B* (3 cases), *TUBB3* (3 cases), and *TUBGCP6* (2 cases). These gene mutations lead to altered microtubule dynamics, disrupting radial migration of neurons and showing a clear genotype–phenotype correlation. Variations in the *DCX* gene (3 cases) were found in both pachygyria and lissencephaly phenotypes, demonstrating its multifaceted regulatory role in neuronal migration. The *PAFAH1B1* gene is primarily detected through chromosomal deletion (17p13.3), but it also plays a crucial role at the single-gene level. Additionally, mutations in the *FLNA* gene (5 cases) are associated with classic subependymal nodular heterotopia and have also been identified in isolated gyral abnormalities, thereby expanding its phenotypic spectrum. Particularly noteworthy is that mutations in mTOR pathway-related genes, such as *TSC2* (2 cases), are associated not only with cortical nodules in tuberous sclerosis but also detected in isolated gyral abnormalities, reflecting the critical role of signaling pathways in the migration process. Mutations in extracellular matrix-related genes such as *POMT2* and *FKTN* lead to cobblestone-like malformations by affecting the integrity of the basement membrane, thereby further enhancing our understanding of the molecular mechanisms behind migration abnormalities.

#### 5.2.4. Molecular Basis of Complex MCD: The Role of Cross-Developmental Stage Regulatory Genes

Complex MCDs involve abnormalities in multiple developmental stages, making their genetic mechanisms particularly complex. In these cases, we identified multiple genes with functions spanning across developmental stages. Mutations in the *ASPM* gene not only cause isolated microcephaly but have also been identified in a compound phenotype of microcephaly combined with cortical gyral abnormalities (8 cases), suggesting its role in both neural progenitor cell proliferation and neuronal migration. Similarly, mutations in the *TUBB2B* gene not only cause migration-phase abnormalities but are also found in a compound phenotype of microcephaly with lissencephaly, illustrating the dual role of tubulin proteins in both cell division and neuronal migration. Mutations in the *AKT1* gene were found in cases of megalencephaly combined with lissencephaly, polymicrogyria, and heterotopia, demonstrating the central role of the PI3K-AKT-mTOR pathway in coordinating multiple developmental processes.

### 5.3. Comparison of Prenatal Genetic Diagnostic Rates

Among the 58 studies included, 7 cohort studies provided detailed diagnostic performance data (Table 2), encompassing the application of various detection techniques across different MCD phenotypes. By analyzing these studies, we explored how technology selection and phenotypic complexity influence diagnostic rates.

Firstly, the diagnostic rates reported by the cohort studies varied significantly, mainly influenced by detection technology, phenotypic complexity, and cohort sample characteristics. Comparative data indicate that CMA had a low diagnostic rate for some phenotypes, such as microcephaly. For instance, in the study by Sukenik-Halevy et al. [16], CMA showed a diagnostic rate of only 4.60% for microcephaly. However, when a combined approach using CMA, WES, and WGS was applied, the diagnostic rate significantly improved, particularly for polymicrogyria and microcephaly. For instance, in the study by Wang LL et al. [15], the combination of CMA and WES resulted in a diagnostic rate of 68.75%, demonstrating that WES and WGS provide more precise genetic information, thus significantly improving diagnostic efficiency.

Secondly, diagnostic rates for different MCD phenotypes varied, depending on both the detection technology employed and the genetic heterogeneity of the phenotypes themselves. For example, in some cohorts using only CMA, the diagnostic rate for microcephaly was extremely low (e.g., 1.89% in the study by Pasternak Y et al. [17]), highlighting the limitations of CMA in diagnosing these complex phenotypes. In contrast, WES/WGS showed significantly better diagnostic performance. In the microcephaly cohort, incorporating WES increased the diagnostic rate from under 5% with CMA to 16.96–23.56% [14,18]. Notably, in the megalencephaly phenotype, the diagnostic rate for WES reached 39.08%, mainly due to its efficient detection of single-nucleotide variations in key signaling pathways like PI3K-AKT-mTOR [13]. WGS has been used less frequently in the existing cohort, but it theoretically offers broader genome coverage compared to WES [55,76].

Genetic pattern analysis of 237 pathogenic variants showed that 120 cases (50.6%) were de novo mutations; 50 cases (21.1%) followed an autosomal recessive (AR) inheritance pattern, inherited from carrier parents with normal phenotypes; 8 cases (3.4%) followed X-linked recessive (XL) inheritance, affecting male fetuses (mutations inherited from the mother); 10 cases (4.2%) followed autosomal dominant (AD) inheritance (1 case inherited from a mother with epilepsy, hydrocephalus, and intellectual disability; 6 cases from parents with no phenotype; and 3 cases with unknown parental origin); and 49 cases (20.7%) had an unknown inheritance pattern. It is evident that de novo mutations are the primary genetic mechanism in prenatal MCD. Notably, a total of 178 cases (75.1%) of pathogenic variants (including all de novo mutations, AR, and XL mutations) cannot be detected by routine prenatal screening. These mutations are often not associated with a clear family history (such as AR carrier parents with normal phenotypes or XL carrier mothers with mild or normal phenotypes), making their genetic risk easily underestimated in prenatal screening. Without specific genetic testing, these causes are difficult to detect through routine imaging screening. Therefore, for fetuses with prenatal imaging indicating MCD, traditional screening methods alone are insufficient to fully assess their genetic etiology and recurrence risk. Prenatal genetic testing is necessary to clarify the pathogenic mechanisms, evaluate the prognosis, and provide accurate genetic counseling and reproductive guidance to the family.

Within the current research framework, the diagnostic value of various testing techniques has been initially established. For MCD fetuses, the diagnostic effectiveness of CMA is limited because many of the genetic causes involve single-gene mutations that CMA cannot identify. It is important to note that rapid technological advancements are blurring the traditional distinction between WES and CMA. The current bioinformatics pipeline can reliably detect clinically significant copy number variants (CNVs) using WES data. In the cases included in this study, WES successfully identified pathogenic CNVs, such as the microdeletions at 17p13.3 and 7q11.23 [14]. Currently, WES has gradually become a routine first-line diagnostic tool for pediatric neurological abnormalities such as cerebral palsy, intellectual disabilities, and epilepsy [83]. Therefore, advancing the postnatal validated high-level diagnostic techniques to the prenatal stage is an inevitable step towards achieving early and precise diagnoses. Although the traditional “CMA-first, WES-second” stepwise strategy is cost-effective, it may result in extended diagnostic timelines. For prenatal diagnoses with limited gestational windows (especially since many MCD phenotypes manifest in late pregnancy), such delays can have significant consequences. While CMA still offers certain advantages in detecting low-level mosaicism and trisomy, WES provides a more comprehensive and efficient diagnostic solution in most cases. Considering that the etiology of MCD (especially complex types) is predominantly caused by single-gene mutations, prenatal WES is expected to become the first-line diagnostic tool for MCD and other fetal neurological abnormalities.

However, genetic diagnostic cohort studies on prenatal MCD are still in the preliminary stages, with limitations primarily in two areas. First, there is a limited number of high-quality cohort studies, most of which are focused on single centers, resulting in inadequate sample sizes and statistical power. Second, existing studies exhibit a clear imbalance in phenotype selection. As shown in Table 2, the majority of studies focus on head circumference abnormalities (microcephaly or macrocephaly), which are emphasized due to their ease of screening and measurement in routine prenatal ultrasound. In contrast, there is a severe lack of cohort studies that systematically evaluate genetic factors in more complex cortical structural abnormalities, such as cortical gyral malformations (e.g., lissencephaly, polymicrogyria) or gray matter heterotopia. This has led to a limited understanding of the genetic basis of these specific phenotypes, and the true diagnostic rates may be skewed, which hinders precise clinical guidance.

## 6. Prenatal Seizure-like Activity as an Early Functional Signal in MCD

Seizure-like activity observed during fetal life is exceptionally rare but may indicate severe functional impairment of the fetal central nervous system, warranting special attention in the prenatal evaluation of MCD. Importantly, fetal seizures are not formally recognized as a diagnostic entity by the International League Against Epilepsy (ILAE) and should therefore be interpreted in the prenatal context as a phenomenological description based on behavioral and dynamic imaging observations rather than a definitive diagnosis of epilepsy [84,85].

Reported cases of seizure-like fetal activity are most often identified in mid-to-late gestation, typically after 20 weeks of gestation, either through maternal perception of abnormal fetal movements or by real-time ultrasound demonstrating repetitive, rhythmic, and paroxysmal movement patterns [85,86]. This gestational distribution has been attributed to the progressive maturation of fetal brain networks and the emergence of cortical excitability necessary for epileptiform activity. However, due to the absence of objective electrophysiological assessment in utero, distinguishing seizure-like activity from non-epileptic fetal movements—such as benign hyperactivity, myoclonus, hiccups, cord-related movements, or early manifestations of neuromuscular disorders—remains challenging, and misclassification has been acknowledged across published series [85].

From an etiological perspective, seizure-like fetal activity has been reported in a heterogeneous spectrum of conditions, including hypoxic–ischemic brain injury, severe central nervous system malformations, metabolic disorders, fetal akinesia-related conditions, and genetic epileptic encephalopathies. More recent case reports have expanded this spectrum by demonstrating that ion channel-related epileptic encephalopathies, such as *SCN8A*- and *SCN2A*-associated disorders, may manifest with seizure-like activity already in utero, frequently in association with major structural brain abnormalities detected on prenatal imaging [87,88]. Although these observations are largely based on isolated cases, they consistently suggest a high risk of adverse neurological outcome and a strong likelihood of de novo pathogenic variants.

In the specific context of MCD, epilepsy typically emerges postnatally as a dominant clinical feature. Nevertheless, the prenatal occurrence of seizure-like activity may indicate not only structural cortical dysgenesis but also the early establishment of epileptogenic networks or the coexistence of functional hyperexcitability disorders, thereby compounding disease severity. From a clinical standpoint, such findings should be regarded as high-risk neurological signals, prompting expedited reassessment with targeted neurosonography and fetal MRI, early comprehensive genetic testing with WES as a core approach, and careful perinatal planning at centers equipped for advanced neonatal neurological and epilepsy care.

## 7. Conclusions and Outlook

This review provides a systematic overview of the imaging phenotypic spectrum, genetic etiology, and prenatal diagnostic strategies for fetal MCD. Through the integration of current evidence, we have highlighted that the phenotype of prenatal MCD is distinctly clustered in developmental stages, with proliferation-phase abnormalities predominating. Importantly, high-throughput sequencing technologies, particularly WES, have demonstrated significant potential in addressing the high genetic heterogeneity of MCD, progressively solidifying their pivotal role in prenatal precision diagnosis. We found that de novo mutations are the primary genetic pattern in prenatal MCD, and the vast majority of pathogenic mutations remain undetectable through routine screening, emphasizing the need for proactive genetic testing in fetuses with abnormal imaging findings.

While high-throughput sequencing technologies, especially whole-exome sequencing, have substantially advanced our understanding of the marked genetic heterogeneity of MCD, they should not be regarded as standalone solutions for all diagnostic challenges. The diagnostic yield of genetic testing remains incomplete, with a considerable proportion of cases lacking an identifiable pathogenic variant, thereby limiting prognostic accuracy and genetic counseling. In addition, the interpretation of variants of uncertain significance requires cautious, case-by-case evaluation within an appropriate clinical context.

Future research on fetal MCD should therefore emphasize an integrated and transdisciplinary diagnostic paradigm. Establishing a truly multidisciplinary framework that combines fetal imaging, clinical genetics, fetal neurology, metabolic assessment, and longitudinal follow-up is essential for achieving early and accurate diagnosis. Underexplored yet clinically relevant areas—such as the relationship between fetal seizures and MCD, as well as inborn errors of metabolism presenting with cortical malformations—deserve further systematic investigation.

Moreover, large-scale, prospective, multicenter cohort studies are urgently needed to bridge the knowledge gap in complex phenotypes such as cortical gyral malformations and to build a more comprehensive genotype–phenotype–prognosis correlation map. As multi-omics approaches and functional validation systems continue to evolve, a more comprehensive understanding of MCD pathogenesis is anticipated. Ultimately, such progress will support more precise genetic counseling, informed reproductive decision-making, and the development of early intervention strategies for affected families.

## Figures and Tables

**Table 1:** Distribution of phenotypes in fetal malformations of cortical development.

Classification	Phenotype	Number	Total
Type I	Microcephaly	107	149
	Macrocephaly	39	
	Megalencephaly	2	
	Hemimegalencephaly	1	
Type II	Pachygyria–lissencephaly	22	61
	Abnormal gyration/sulcation	26	
	Polymicrogyria	7	
	Heterotopia	3	
	Others	3	
Type III	Focal cortical dysplasia	3	3
Type I + II	Microcephaly + Abnormal gyration	9	24
	Macrocephaly + Abnormal gyration	8	
	Megalencephaly + Abnormal gyration	2	
	Microlissencephaly	5	

**Table 2:** Summary of cohort studies on the diagnostic efficacy of genetic diagnosis for fetal malformations of cortical development.

First Author	PMID	Year	Country	Imaging Findings	Technique	Number of Cases Included (*N*)	Number of Positive Diagnoses (*n*)	Detection Rate (*n*/*N*)
Zhou H [13]	40404351	2025	China	Macrocephaly	WES	87	34	39.08%
Chen J [19]	39701139	2025	China	MCD	CMA/WES/WGS	95	31	32.63%
Wang LL [15]	38634212	2024	China	MCD	CMA/WES	32	22	68.75%
Sukenik-Halevy R [16]	38494511	2024	Israel	Microcephaly	CMA	87	4	4.60%
Liu J [18]	38113582	2024	China	Microcephaly	CMA/WES	157	37	23.56%
Wang Y [14]	37229200	2023	China	Microcephaly	CMA/WES	224	38	16.96%
Pasternak Y [17]	32721143	2020	Israel	Microcephaly	CMA	53	1	1.89%

## Data Availability

No new data were created or analyzed in this study. Data sharing is not applicable to this article.

## Supplementary Materials

The following supporting information can be downloaded at: https://www.mdpi.com/article/10.3390/biomedicines14010107/s1. Supplementary Files S1 and S2.

## Abbreviations

The following abbreviations are used in this manuscript:

MCD	Malformations of cortical development
CMA	chromosomal microarray analysis
WES	whole-exome sequencing
WGS	whole-genome sequencing
FCD	focal cortical dysplasia
FGR	fetal growth restriction
CNV	copy number variants
XL	X-linked recessive
