# Supplementary material for: Prenatal Diagnosis of Malformations of Cortical Development: A Review of Genetic and Imaging Advances
Source: Biomedicines. 2026 Jan 5;14(1):107. doi: 10.3390/biomedicines14010107 (PMC12838827; doi:10.3390/biomedicines14010107)
Supplement: Supplementary file 1 [file biomedicines-14-00107-s001.zip › Supplementary File S2.pdf]

Supplementary File S1

Study Selection and Data Extraction

A systematic search was conducted in PubMed, Scopus, and Web of Science up to October 17, 2025, to identify studies on MCD, CMA, or next-generation sequencing (NGS). The search used the following Boolean operators: (("Nervous System Malformations" OR "Central Nervous System" OR "central nervous system malformation*" OR "central nervous system abnormalit*" OR "nervous system malformation*" OR "CNS malformation*" OR "CNS abnormalit*" OR "fetal nervous system" OR "fetal brain abnormalit*" OR "malformations of cortical development" OR MCD OR "cortical malformation*" OR microcephaly OR megalencephaly OR hemimegalencephaly OR "focal cortical dysplasia" OR FCD OR "FCD type I" OR "FCD type III" OR "periventricular nodular heterotopia" OR PVNH OR "subcortical band heterotopia" OR SBH OR heterotopia OR lissencephaly OR agyria OR pachygyria OR "cobblestone lissencephaly") AND (prenatal OR antenatal OR fetal OR fetus OR "in utero" OR "Prenatal Diagnosis") AND ("exome sequencing" OR "whole exome sequencing" OR WES OR "genome sequencing" OR "whole genome sequencing" OR WGS OR "chromosomal microarray analysis" OR CMA OR "copy number variant" OR "copy number variation" OR CNV OR "single nucleotide variant" OR SNV OR "point mutation" OR "targeted sequencing" OR "panel sequencing" OR "gene panel" OR "multigene panel" OR "sequencing panel")).

Based on pre-established inclusion and exclusion criteria, two independent reviewers (Jinhua Hu and R.H.) completed the initial screening, blinded to each other.

Any discrepancies in the screening process were resolved through discussion with a third reviewer (Jin Han) until consensus was reached.

The screening process is as follows: (1) First, the titles and abstracts were reviewed, and studies not meeting the criteria were excluded; (2) Subsequently, the full texts of the selected studies were reviewed to finalize the list of included studies. Any disagreements during the screening process were resolved through discussions with a third reviewer (Long Lu) until consensus was reached. The inclusion criteria for studies were: (1) The subjects were fetuses or children diagnosed with MCD in prenatal environments; (2) The study used CMA or next-generation sequencing technologies (Panel sequencing, Exome Sequencing, Genome Sequencing); (3) Clear sequencing data were provided, including identified genes, transcripts, mutation sites, amino acid changes, and ACMG classification. In addition, we excluded review articles, conference abstracts, guidelines, editorials, comments, and opinion articles. However, due to the rarity of MCD and the fact that most existing reports are case studies, case studies were included in this study's screening. Full-text retrieval was performed for all studies that met the inclusion criteria, and eligibility was assessed using a predefined data extraction form. To prevent omissions, references from the included studies were also reviewed, and no missing literature was found. The study selection process is detailed in Figure 1. For studies meeting the inclusion criteria, after full-text review, data extraction was performed using a predefined Excel sheet. The extracted data included the first author, publication year, sequencing method, total number of patients included, number of positive cases, gene locus information, prenatal/postnatal

phenotypes, imaging findings, etc. All extracted data are provided in Supplementary File S1.
